# Thermal Niche Differentiation in the Benthic Diatom *Cylindrotheca closterium* (Bacillariophyceae) Complex

**DOI:** 10.3389/fmicb.2019.01395

**Published:** 2019-06-21

**Authors:** Willem Stock, Bart Vanelslander, Franziska Rüdiger, Koen Sabbe, Wim Vyverman, Ulf Karsten

**Affiliations:** ^1^Laboratory of Protistology and Aquatic Ecology, Department of Biology, Ghent University, Ghent, Belgium; ^2^Institute of Biological Sciences, Applied Ecology and Phycology, University of Rostock, Rostock, Germany

**Keywords:** *Cylindrotheca closterium*, benthic diatom, thermal niche, ocean warming, cryptic species, niche conservation

## Abstract

Coastal waters are expected to undergo severe warming in the coming decades. Very little is known about how diatoms, the dominant primary producers in these habitats, will cope with these changes. We investigated the thermal niche of *Cylindrotheca closterium*, a widespread benthic marine diatom, using 24 strains collected over a wide latitudinal gradient. A multi-marker phylogeny in combination with a species delimitation approach shows that *C. closterium* represents a (pseudo)cryptic species complex, and this is reflected in distinct growth response patterns in terms of optimum growth temperature, maximum growth rate, and thermal niche width. Strains from the same clade displayed a similar thermal response, suggesting niche conservation between closely related strains. Due to their lower maximum growth rate and smaller thermal niche width, we expect the polar species to be particularly sensitive to warming, and, in the absence of adaptation, to be replaced with species from lower latitudes.

## Introduction

Coastal waters have warmed during the last decades, and are predicted to continue to warm until the end of this century and beyond, potentially by as much as 2 to 3°C depending on the geographical region (Stocker et al., 2013; Schleussner et al., 2015). The effects are even more severe for benthic species since sediment surface temperature changes occur most intensively due to strong attenuation of solar radiation (Harrison, 1985; Jorgensen and Des Marais, 1986).

Evidence is accumulating that rising temperature affects the performance of coastal species in many regions worldwide. Coral reefs are known to be particularly threatened by global warming (Hughes et al., 2017) since the coral-algal symbiosis is living at its upper thermal limit, and temperature anomalies of just 1–2°C above mean local summer maxima are sufficient to cause massive coral bleaching leading to high mortality (Lesser, 2011). Also in temperate regions, warming coastal waters affect temperature-sensitive species such as the brown alga *Fucus vesiculosus* in the Baltic and the North Sea (e.g., Graiff et al., 2015). Coastal microalgae on the other hand might be more resistant to global warming (Woelfel et al., 2014a, 2014b). To accurately predict the effects of coastal warming, a better understanding of how temperature and temperature variations will affect coastal marine organisms is required.

Diatoms are a large and often dominant constituent of the coastal microalgal community (Underwood and Kromkamp, 1999; Malviya et al., 2016). Diatoms exist as benthic and pelagic forms and are regarded as one of the largest and ecologically most successful groups of microorganisms on Earth. They are the most diverse group of marine phytoplankton (Armbrust, 2009). Apart from dominating intertidal mudflats and shallow water coastal zones, diatoms are at the base of the coastal trophic food webs (Cahoon et al., 1999; Underwood and Kromkamp, 1999).

A recent modeling study on *Fragilariopsis kerguelensis*, a dominant diatom species throughout the Antarctic Circumpolar Current and one of the main drivers of the biological silicate pump, indicates that ocean warming might indeed affect the biogeography of this taxon (Pinkernell and Beszteri, 2014). Consequently, rising ocean temperature has the potential to alter the composition and the productivity of marine diatom communities, thereby affecting global biogeochemical cycles (Thomas et al., 2012). Predicting the effects of future ocean warming on biogeochemical cycles of carbon, nitrogen, phosphorus, and silicate depends on understanding how existing global temperature variation affects the important marine primary producers (Laufkötter et al., 2015; Roxy et al., 2016).

Although the decisive ecological roles of diatoms are broadly recognized, knowledge about their biodiversity, geographical distribution and possible endemism on different spatial and temporal scales remains limited, particularly for benthic forms. Morphology-based studies have led to the assumption that many marine diatoms are cosmopolitan and ubiquitous (Cermeño and Falkowski, 2009; Rad-Menéndez et al., 2015), but detailed molecular studies revealed that many of these alleged cosmopolitan diatoms actually consist of several morphologically identical (cryptic) or almost identical (pseudocryptic) species (Kooistra et al., 2008; Casteleyn et al., 2010; Degerlund et al., 2012). This raises the question whether the supposed ecological plasticity of many cosmopolitan species in fact reflects phenotypic differences between geographically restricted cryptic species. The recognition of cryptic diversity might even explain apparently meaningless patterns in the biology or biogeography of species (Amato et al., 2007). Particularly the importance of geography (Casteleyn et al., 2010) and environment (Kooistra et al., 2008) on the structuring of diatom communities requires further investigation when we want to correctly predict the effects of global change (Usinowicz and Levine, 2018).

In addition to the ecological plasticity of diatoms, the evolutionary plasticity of niche characteristics, such as an optimal temperature range, will also be important in predicting species responses to ocean warming (Chivers et al., 2017). The retention of niche characteristics over generations, i.e., niche conservatism, might constrain adaptation in a rapidly changing environment (Pyron et al., 2015). For species living closely to their upper thermal limit and with limited dispersal possibilities, niche conservatism may result in local extinctions (Deutsch et al., 2008; Soininen et al., 2018). Based on field observations, diatoms are thought to not show high niche conservatism (Chivers et al., 2017; Soininen et al., 2018). However, cryptic diversity might be obscuring patterns.

In the present study, *Cylindrotheca closterium* (Ehrenberg) Reimann and Lewin, 1964 was chosen as an ecologically important cosmopolitan benthic diatom species. *C. closterium* is widely distributed in high and low latitude marine to brackish water regions where this species can reach high densities (de Brouwer et al., 2005; Najdek et al., 2005) and also occurs inside sea-ice (von Quillfeldt et al., 2003). An internet search with the Ocean Biogeographic Information System (OBIS^1^ (accessed on August 6, 2018)) resulted in over 30,000 records for this taxon worldwide, indicating a cosmopolitan distribution. However, whether all these records indeed represent the same taxon is unclear since detailed molecular-taxonomical data for most of the samples are missing. Nevertheless, *C. closterium* has been widely used as a diatom model system to study diatom ecophysiology, including the production and function of extracellular polymeric substances (de Brouwer et al., 2005; Pletikapić et al., 2011), movement (Apoya-Horton et al., 2006; Araújo et al., 2013) and anti-oxidative defense (Rijstenbil, 2005).

The main goal of this study was to comprehensively investigate the phylogenetic position and origin of 24 *C. closterium* strains in relation to their thermal response. The *C. closterium* strains were collected from tropical, temperate and polar coastal regions and we thus expected to observe pronounced differences in their thermal growth responses. We further anticipated a phylogenetic signal to be present in their thermal response, allowing us to infer a model on their temperature niche evolution and predicting the future impact of global change on the biogeography of *C. closterium*.

## Materials and Methods

### Isolation and Culturing

**FIGURE 1:**
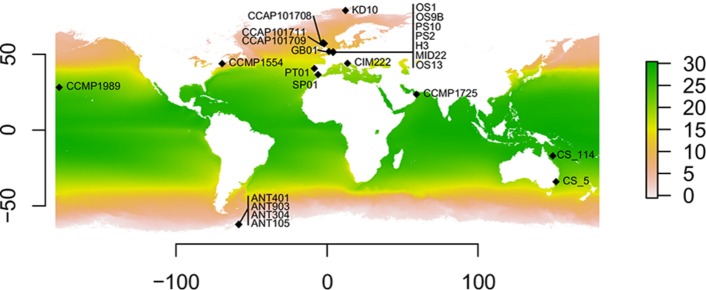
Geographic distribution of the *Cylindrotheca closterium* strains used in this study. Sampling locations with the respective strain names are shown. The average yearly sea surface temperature (in °C) between 2002 and 2018 is indicated by color.

For the present study, 24 strains morphologically corresponding to *C. closterium* s.l. were used (Figure 1). Details on strain number, isolator, biogeographic origin, climatic zone, and geographic location (latitude/longitude) are given in Table 1. Twelve strains were newly isolated from marine and brackish sediment and plankton samples. The strains were isolated from sediments applying the lens tissue technique (Eaton and Moss, 1966), in which migratory behavior was used to collect benthic diatoms by placing a piece of lens tissue on top of the sediment followed by a coverslip on the tissue, which was transferred to autoclaved seawater after 3 h of incubation at low light. From this migrated cell population monoclonal cultures of *C. closterium* were established by isolating single cells using a micropipette followed by subsequent culturing in filtered (0.2 μm) seawater (salinity: 33 S_P_) enriched with f/2 nutrients (Guillard, 1975). The other 12 strains of *C. closterium* were obtained from the National Center for Marine Algae and Microbiota (NCMA), United States; the Commonwealth Scientific and Industrial Research Organization (CSIRO) collection of living microalgae (Australia), and the culture collection of algae and protozoa (CCAP), United Kingdom. Strains PT01, SP01, GB01, and CIM222 were kindly provided by J. Serôdio, I. Moreno Garrido, J. Taylor, and M. Pfannkuchen, respectively.

**TABLE 1:** List of *Cylindrotheca closterium* strains studied.

**Strain number**	**Isolator**	**Date**	**Biogeographic origin**	**Climate zone**	**Location**	**Yearly average SST (°C)**
*CS-114*	J.L. Stauber	1980	Coral Sea, Australia	Tropical	17° S, 149° E	26.5
*CCMP1725*	J. Stirn	1995	Gulf of Oman, Arabian Sea	Tropical	23° 34′ N, 58° 51′ E	27.3
*CCMP1989*	N. Rolde	1997	Midway Islands, United States	subtropical	28° 12′ N, 177° 21′ W	23.5
*CS-5*	M. Wotten	1962	Port Hacking, Australia	subtropical	34° 04′ S, 151° 08′ E	19.3
*SP01*	I.Moreno-Garrido	2000	Puerto Real, Spain	subtropical	36° 36′ N, 6° 12′ W	19.1
*PT01*	J. Serôdio	2004	Rio de Aveiro, Portugal	subtropical	40° 39′ N, 8° 40′ W	15.0
*CIM222*	M. Pfannkuchen	2009	Adriatic Sea, Croatia	subtropical	44° 02′ N, 13° 14′ E	18.1
*CCMP1554*	D. Jacobson	1993	Boothbay Harbor, Maine, United States	Temperate	43°50′N, 69° 38′ W	9.7
*PS10*	B. Vanelslander	2008	Western Scheldt, Netherlands	Temperate	51°21′ N, 3°43’ E	11.9
*PS2*	B. Vanelslander	2008	Western Scheldt, Netherlands	Temperate	51°21′ N, 3°43′ E	11.9
*OS13*	B. Vanelslander	2007	Eastern Scheldt, Netherlands	Temperate	51°32′N, 3°44′ E	12.9
*OS9B*	B. Vanelslander	2007	Eastern Scheldt, Netherlands	Temperate	51°32’N, 3°44′ E	12.9
*OS1*	B. Vanelslander	2007	Eastern Scheldt, Netherlands	Temperate	51°32′N, 3°44′ E	12.9
*MID22*	B. Vanelslander	2007	Veerse Meer, Netherlands	Temperate	51°33′N, 3°47′ E	11.8
*H3*	B. Vanelslander	2007	Western Scheldt, Netherlands	Temperate	51°21′ N, 3°43′ E	11.9
*GB01*	J. Taylor	2008	Colne, United Kingdom	Temperate	51° 50′ N, 0° 59′ E	12.1
*CCAP101711*	E. Bresnan	2004	Stonehaven, Scotland, United Kingdom	Temperate	56° 58′ N, 2° 12′ W	9.3
*CCAP101709*	E. Bresnan	2004	Cove Bay, Scotland, United Kingdom	Temperate	57° 06′ N, 2° 04′ W	9.4
*CCAP101708*	E. Bresnan	2004	Buckie, Scotland, United Kingdom	Temperate	57° 40 N, 2° 58′ W	9.6
*ANT105*	B. Vanelslander	2010	King George, Antarctica	Cold	62°13′ S, 58°40′ W	0.4
*ANT304*	B. Vanelslander	2010	King George, Antarctica	Cold	62°13′ S, 58°40′ W	0.4
*ANT401*	B. Vanelslander	2010	King George, Antarctica	Cold	62°13′ S, 58°40′ W	0.4
*ANT903*	B. Vanelslander	2010	King George, Antarctica	Cold	62°13′ S, 58°40′ W	0.4
*KD10*	J. Woelfel	2005	Ny-Ålesund, Spitsbergen. Norway	Cold	78° 55′ N, 11 56′ E	1.5

*Given are strain number, isolator, biogeographic origin, climatic zone, geographic location, coordinates and the yearly average sea surface temperature (SST) of that location. Strains are ordered according their biogeography from low to high latitudes.*

All stock cultures, except the polar strains, were kept in 24-well plates (Greiner Bio-One, Frickenhausen, Germany) at 18 ± 0.3°C with a 16:8 h light: dark period and 50 μmol photons m^−2^s^−1^ provided by cool-white fluorescent tubes (Philips TLD 18W, Philips Ltd., Eindhoven, Netherlands) in the weeks prior to the experiment. Originally, the CCAP were maintained at 15°C, CS-114 at 25°C, the (sub)tropical CCMP strains at 24°C and the temperate CCMP strain at 14°C. The polar strains ANT105, ANT304, ANT401, ANT903, and KD10 were isolated and maintained at 6°C, under identical light conditions. All cultures were transferred every 2 weeks to fresh medium.

### Genetic Identity and Phylogeny

The evolutionary history of the 25 *Cylindrotheca* strains (24 *C. closterium* and one *C. fusiformis* as outgroup) strains was inferred from a multi-locus DNA dataset. Five DNA regions were selected, which included the nuclear ITS region (consisting of ITS1, 5.8S rRNA gene, and ITS2) and D1/D2 region of the LSU rRNA gene, the chloroplast RuBisCO large subunit gene (*rbcL*) and *psbA* and the mitochondrial gene *cox*1. Cells for DNA extraction were harvested from exponentially growing cultures and pelleted by centrifugation. DNA was extracted using the bead-beating method with phenol extraction and ethanol precipitation according to Zwart et al. (1998). After extraction, DNA was purified with a Wizard© DNA Clean-up system (Promega). PCR products were obtained using previously published PCR primers and protocols *rbcL* and ITS: Vanelslander et al. (2009), *cox*1: Evans et al. (2007), LSU: Souffreau et al. (2013), *psbA*: Souffreau et al. (2011). PCR products were cleaned using QIAquick PCR Purification Kit (Qiagen, Hilden, Germany) following the manufacturer’s instructions. The sequencing reactions were performed by cycle sequencing (initial step of 1 min at 96°C, 30 cycles of 10 s at 96°C, 10 s at 50°C, and 1 min 15 s at 60°C) using the ABI Prism Big-Dye V 3.1 Terminator Cycle Sequencing kit (Applied Biosystems). The resulting sequencing reaction products were analyzed on a Perkin–Elmer ABI Prism 3100 automated DNA sequencer (Applied Biosystems). All sequences newly generated during this study were deposited in GenBank (accession numbers LSU: MH704526- MH704550; ITS: MH716187- MH716211, *rbcL*: MH807634-MH807658, *psbA*:MH819197- MH819219, *cox*1:MH819220- MH819235).

The sequences were aligned using ClustalW (Mega 7.0.14) under standard settings followed by manual curation and trimming of low-quality regions. The sequences for the different loci were concatenated in sequenceMatrix (1.8). This resulted in an alignment of 4182 nucleotide positions: 575 for *cox*1, 797 for ITS, 525 for LSU, 828 for *psbA* and 1454 for *rbcL*. The concatenated data matrix was incomplete for *cox*1, which was missing for 8 strains and *psbA* which was missing for 2 strains. MrModeltest 3.7 (Nylander, 2004) was used to establish the most appropriate model of DNA evolution (GTR+I+G), which was used in subsequent phylogenetic reconstructions. A maximum likelihood (ML) phylogeny was constructed with RAxML (Stamatakis, 2014) under default settings with the *C. fusiformis* strain as the outgroup and the alignment partitioned into the different loci. Additionally, single locus ML trees were constructed in RAxML using identical settings. Bayesian phylogenetic inference (BI) was performed using MrBayes version 3.2.6 (Ronquist and Huelsenbeck, 2003). The GTR+I+G model was used in which each protein-coding gene (*cox*1, *psbA*, and *rbcL*) was partitioned into three codon positions and LSU and ITS were treated as separate partitions, resulting in a total of 11 partitions. The *C. fusiformis* strain was again set as the outgroup. All parameters were unlinked between partitions. Two independent runs of three heated and one cold Metropolis-coupled Monte-Carlo Markov Chains (MCMC) were run for 30 million generations using default settings and with a relative burn-in set. Runs were sampled every 1,000th generation. Convergence and stationarity of the log-likelihood and parameter values was assessed using Tracer v.1.5 (Rambaut and Drummond, 2009) after which a relative burn-in of 25% was used.

For species delimitation, two maximum likelihood phylogenies were constructed based on the *rbcL* and ITS sequences. Using only the unique haplotypes and no outgroup, RAxML was used with the settings identical as those used for constructing the concatenated phylogeny. The Poisson tree processes (PTP) model was used to infer putative species boundaries on these ML trees (Zhang et al., 2013). The bPTP server (Zhang et al., 2013) was used to obtain both ML and BI based PTP search results for both phylogenies.

### Growth Measurements

The temperature requirements for growth in all 24 strains of *C. closterium* and the *C. fusiformis* strain were assessed by culturing at a range of different temperatures (0.5, 5, 10, 15, 20, 25, and 33°C). Each temperature treatment was replicated four times and all strains were acclimated for 4 weeks to the experimental temperatures before growth was evaluated. Cells for growth experiments were always harvested from exponentially growing cultures and inoculated in 24-well plates at a cell density of ∼3,000 cells ml^−1^. Light conditions and culture medium were identical to those described above for the stock cultures. Growth was monitored by pulse amplitude modulated (PAM) fluorometry (MAXI Imaging PAM Fluorometer, Walz, Germany) according to the principal methodological approach of Gustavs et al. (2009). The minimum fluorescence yield F_0_ was used as a proxy for biomass (Honeywill et al., 2002). The growth rate was determined during the exponential growth phase (4–5 days) as the slope of the linear regression of log2-transformed F_0_ fluorescence versus time for individual cultures. The growth rate averaged over the respective replicates was used for further analyses. All the hereafter mentioned analyses were run in R version 3.4.1. Graphics were constructed using ggplot 2 (version 2.2.1).

The relation between growth rate and temperature was modeled according to Blanchard et al. (1996) using the following function:

$$\mu \,\left(T\right)=\mu_{m\,a\,x}\,{\left(\frac{T_{m\,a\,x}-T}{T_{m\,a\,x}-T_{o\,p\,t}}\right)}^{\beta }\,e\,x\,p\,\left[-\beta \,\left(\frac{T_{m\,a\,x}-T}{T_{m\,a\,x}-T_{o\,p\,t}}-1\right)\right]$$

μ*_max_*: maximum growth rate per day (μ d^−1^)

T: temperature

T*_opt_*: optimum temperature for growth

T*_max_*: maximum temperature for growth

β: dimensionless parameter, describing the slope of the growth curve.

The parameters of the growth equation were estimated by a non-linear least squares algorithm (nlsLM; minpack.lm -version 1.2; Elzhov et al., 2010). The standard errors for T*_opt_* and T*_max_* were calculated by the summary.nls.lm function (minpack.lm package). The relation between T*_opt_* and μ*_max_*, as predicted by the model, was evaluated in a linear regression whereby μ*_max_* was expressed in function of T*_opt_*. In comparison, the relation of maximally observed growth (observed μ*_max_*) to the experimental temperature, at which this growth had occurred, was also assessed with a linear regression.

The thermal performance range of each strain, defined as the temperature range at which ≥80% of the predicted maximum growth (performance) can be achieved (Huey and Stevenson, 1979), was calculated using the rootSolve package (version 1.7; Soetaert, 2009).

#### Phylogenetic Signal

A maximum likelihood consensus tree was converted to an ultrametric tree using penalized likelihood rate smoothing with a lambda value of 0.1 using the ape package (version 5.1; Sanderson, 2002). The correlation between the phylogenetic position of the strains and their predicted thermal optimum was evaluated using Pagel’s λ (Pagel, 1999) and Blomberg’s K (Blomberg et al., 2003). The parameters were estimated and corresponding hypothesis tests were performed using Phylotools (version 0.6; Revell, 2012).

The similarities between strain phylogeny and geographical origin were tested. The genetic distances between strains (phylogeny) were calculated as the pairwise proportion of nucleotide sites at which the *rbcL* sequences, the largest marker, differ between strains using Mega7 (Kumar et al., 2016). The geographical distances between strains were calculated based on the coordinates provided in Table 1. A mantel test (ade4 version 1.7; Dray and Dufour, 2007) was used to test if there was a relationship between the genetic and the geographical distance matrices.

### Viability After Incubation at Extreme Temperatures

For a subset of *C. closterium* strains which did not show growth at 0.5°C or 33°C, cell viability was evaluated after 4 weeks incubation at the respective temperature using the fluorescent dye SYTOX-Green. Cells with intact cell membranes were distinguished from those with permeabilized membranes, using the nucleic acid stain SYTOX-Green according the protocol of Veldhuis et al. (2001). SYTOX-Green can only pass through compromised or damaged membranes, and stains the nucleus leading to enhanced fluorescence under blue light excitation. Consequently, viable *C. closterium* cells can be distinguished and quantified from dead cells using epifluorescence microscopy.

Strains CS-114, CS-5, SP01, PT01, CCMP1554, PS10, MID22, and GB01, which did not, or minimally, grew at 0.5°C, were kept for 4 weeks at this temperature. To test their tolerance at higher temperature, strains KD10, GB01, H3, MID22, OS1, OS13, PS2, and SP01, which did not, or minimally, grew at 33°C, were maintained at this enhanced temperature. Media and light conditions were identical to those of the other growth experiments already described above. Media were refreshed every second week.

After 4 weeks of treatment, cells were harvested by gentle centrifugation at 1000 × *g* for 2 min (Heraeus Labofuge) and the pellets incubated in one drop of diluted SYTOX-Green (Invitrogen) stock solution for 5 min according the manufacturers protocol (see also Veldhuis et al., 2001). The stained cells were counted under an inverse epifluorescence microscope (IX70, Olympus, Hamburg, Germany) at a magnification of 40 ×. For each of the 3 replicate samples 400 cells were counted as viable or dead.

### Sea Surface Temperatures

The sea surface temperature (SST) was calculated using remote sensing data for the locations from which the strains were isolated. As the locations of interest were generally at the coast, an 8 km buffer around each location was used to prevent the use of data from a mixed ocean/land pixel. The night time skin temperature data, measured at 11 μm band by MODIS-Aqua (NASA OB.DAAC, Greenbelt, MD, United States) was averaged for each season between 2002 and 2018 (the full time window for which these data were available) at a 4 km resolution. The average yearly SST was then calculated by averaging the seasonal SSTs and standard deviation between seasonal SSTs was calculated to represent the seasonal variability in SST at each location. Although these oceanic SSTs do not necessarily best reflect the locally experienced temperatures by the coastal strains (Potts and Swart, 1984), they were considered to reliably reflect the general differences between locations. The SSTs were therefore used as a proxy for the *in situ* conditions. The relation between the average SST and the predicted optimal temperature (T_op_) was assessed with a linear regression.

## Results

### Genetic Identity and Phylogeny

**FIGURE 2:**
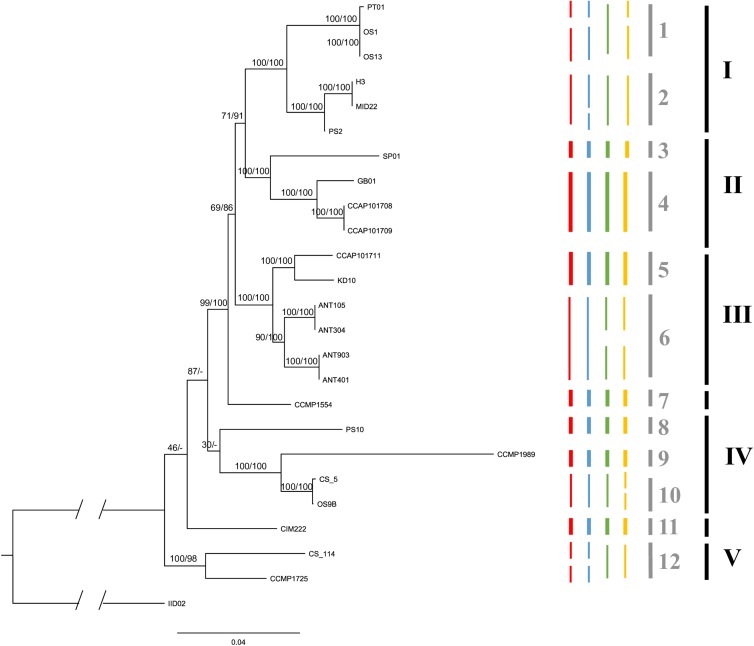
The maximum likelihood consensus tree based on 1000 rapid bootstrap inferences of a concatenated five molecular loci alignment in strains of *C. closterium.* Numbers at nodes are the bootstrap values (ML) and posterior probabilities (BI), respectively, indicating the support for each node; branch lengths represent the expected number of substitutions per site. The truncated part of the outgroup represents an additional 0.22 expected substitutions per site. Clades, indicated by the black bars and roman numerals (see section “Results”) were defined based on similar thermal responses. The colored bars represent the putative species based on different PTP models: red (ML search method) and blue (BI search method) are the putative species delineated using the ITS data, whilst green red (ML search method) and yellow (BI search method) are the putative species based on the *rbcL* data. The species delineation bars are in bold if the four predictions are consistent for that species prediction. The gray bars indicate the conservatively delineated putative species based on the four predications. Each species is indicated with an Arabic numeral.

The phylogenetic analyses, using the five different loci, resulted in a well-supported tree (Figure 2) for the shallow-branching nodes. For deep-branching nodes, support was generally low and appears to have been caused by the topological incongruence of the different markers (Supplementary Material S1).

The different PTP models, used to infer putative species boundaries, suggested the presence of multiple species within the *C. closterium* phylogeny (Figure 2). Based on the ITS phylogeny, 15 and 14 putative species were delineated using the BI and ML search method, respectively. The same approaches resulted in 15 and 13 putative species based on the *rbcL* phylogeny. A conservative consensus estimate, considering only the species delineated by all four methods, resulted in 12 putative species (Figure 2, gray bars). Many of the delineated species were only represented by a single strain.

Despite the clustering of some strains with the same geographical origin (for instance the Antarctic isolates), there was no significant relation between geographical distances and genetic distances of the strains (Mantel test, *p* = 0.07). This can largely be explained by the high genetic diversity found at some of the locations, particularly at the Scheldt in Netherlands.

CCMP1989, a strain from an isolated island (Midway Islands, United States) in the Pacific, is quite distinct from the other strains. This is due to the cumulative effect from unique nucleotide differences in several of the loci. Similarly, another strain from the United States (Maine), CCMP1554 forms a distinct lineage on its own. The same is true for CIM222, a strain originating from the Adriatic Sea.

**FIGURE 3:**
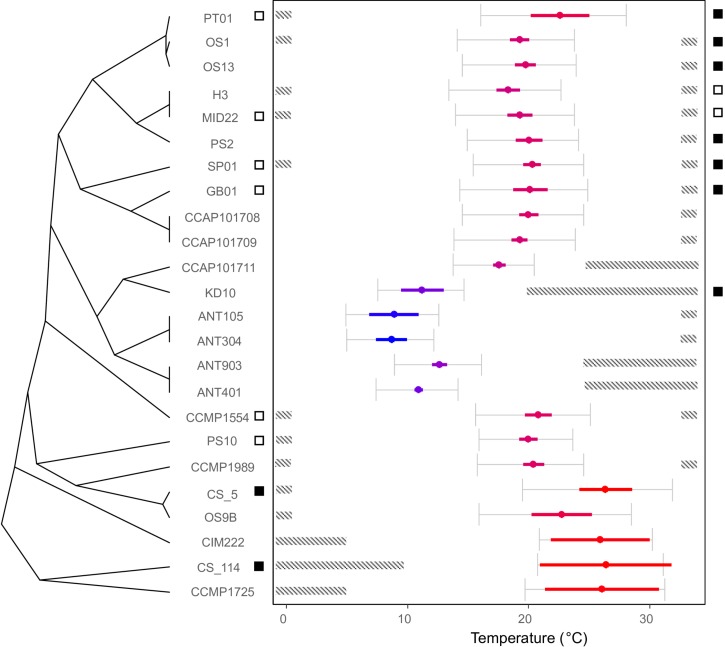
Thermal optima of the *C. closterium* strains relates to their phylogenetic position. The cladogram on the left shows the phylogenetic relations between strains and the estimated optimal temperature is shown by the colored dot ( ± SE) in the temperature plane on the right. The color indicates the relative position of the optimal temperature in relation to the other strains (blue colder to red hotter). The gray error bars indicate thermal performance range (the temperature range at which ≥80% of the predicted maximum growth is estimated) of each strain. The dashed bars indicate areas outside the tolerance range, where no growth was detected. Lastly, the squares on both sides of the temperature plane represent the results for the viability assay: a white square indicates that viable cells were detected in the culture 4 weeks after incubation at 0.5°C (left side) or 33°C (right side), whilst a black square indicates the absence thereof.

Based on the phylogenetic tree, several clades could be delineated (indicated by roman numerals in Figure 2) which contain strains that have similar thermal optima (Figure 3). Each of these clades consists of several putative species. Both Clade I and IV contain temperate strains from Netherlands (Clade I: OS1, OS13, H3, MID22, PS2; Clade IV: PS10, OS9B; Table 1) and subtropical strains (Clade I: PT01; Clade IV: CCMP1989 and CS_5). Similarly, Clade II includes both temperate strains from the United Kingdom (CCAP101708, CCAP101709, and GB01) and a subtropical strain from Spain (SP01). Clade III also contains a strain from the United Kingdom (CCAP101711) as well as strains from colder regions, Spitsbergen, Norway (KD10) and Antarctica (ANT). Lastly, Clade V contains two tropical strains from the Arabian Sea (CCMP1725) and Australia (CS-114).

### Temperature Requirements for Growth

The investigated *C. closterium* strains showed different growth responses to the applied temperature regimes (Supplementary Material S2). None of the isolates grew across the entire temperature range between 0.5 and 33°C (Figure 3). Temperature requirements for growth generally corresponded closely to the biogeographical origin of strains. With the exception of the fifth delineated species (Figure 2), all strains of the same species also showed very similar growth responses to temperature.

Most of the subtropical and tropical strains exhibited growth optima above 20°C and upper tolerance levels of 33°C or higher (Figure 3). In contrast, all polar strains showed optimum growth around 10°C. The temperate strains originated from different habitats between 43°N and 57°N and all grew best around 20°C. Remarkably, for some strains, there was no overlap between thermal performance ranges.

The temperature response of the strains was also compared to their relative phylogenetic position. Two quantitative measures were calculated to express the phylogenetic signal present in the variation observed in the predicted thermal optima: Pagel’s λ (Pagel, 1999) and Blomberg’s K (Blomberg et al., 2003) Both are significantly different from zero (Pagel’s λ = 0.943616 with *p* < 0.0001 and Blomberg’s *K* = 0.02 with *p* = 0.004), indicating that related strains indeed have the tendency to resemble each other’s thermal optimum. Thermal niches thus seem to be, at least, partly conserved within the *C. closterium* complex.

For Pagel’s λ and Blomberg’s K, a value of zero would suggest phylogenetic independence of the thermal response whilst a value of one would indicate that the thermal optimum of species is perfectly distributed along the phylogeny as expected under Brownian motion of trait evolution. Whilst Pagel’s λ is close to one, this is not the case for Blomberg’s K. Blomberg’s K has been shown to be more sensitive to aspects such as the number of strains in the phylogeny (Münkemüller et al., 2012; Molina-Venegas and Rodríguez, 2017). Visual inspection confirms the link between thermal optimum and phylogenetic position (Figure 3) as the strains within clades generally show a similar thermal optimum. There is no clear trend with the sequence of clades as the polar clade is nested within the temperate clades, nor is there a strong link of the thermal optimum with the temperature the strains were maintained at.

**FIGURE 4:**
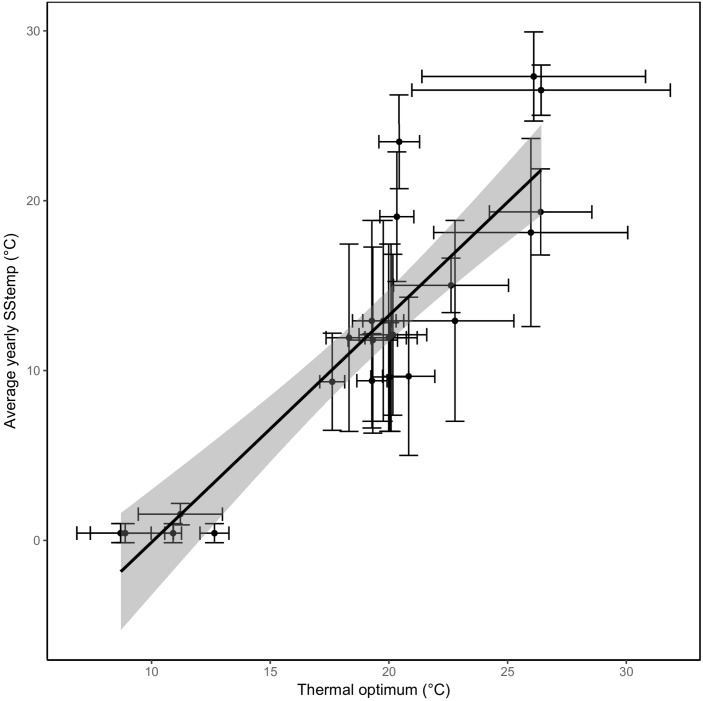
A strong correlation between sea surface temperatures and thermal optima. The predicted optimal temperature (*x*-axis) correlates very well with the average yearly sea surface temperature from where the strains were isolated (*y*-axis). The regression through the data points is shown with the confidence interval shaded in gray. The standard deviation of sea surface temperature based on the seasonal variation and the standard error of the predicted optimal temperature (see section “Materials and Methods”) are represented as error bars.

The predicted thermal optima correlated closely with the average SST from the location they were isolated from (Pearson correlation coefficient of 0.89; *p* < 0.0001, Figure 4). An increase in average SST of 1°C was met with an expected increase of 0.6°C in thermal optimum. Similar relations were found when comparing the different seasonal SSTs to the thermal optima (*r*_Summer_ = 0.89, *r*_Autumn_ = 0.90, *r*_Winter_ = 0.75, and *r*_Spring_ = 0.88).

The thermal performance range width differed between species (Figure 3) and was generally smallest for the polar strains. The width increased approximately with 0.26°C for every 1°C increase in optimal growth temperature of the strains (*p* < 0.0001; Pearson correlation = 0.78). The width correlated marginally (Pearson correlation coefficient of 0.36; *p* = 0.09) with the standard deviation of the SST from where the strains were originally isolated. A stronger positive correlation was found between the thermal performance range width and the predicted maximum growth rate (Pearson correlation coefficient of 0.69; *p* = 0.0002).

**FIGURE 5:**
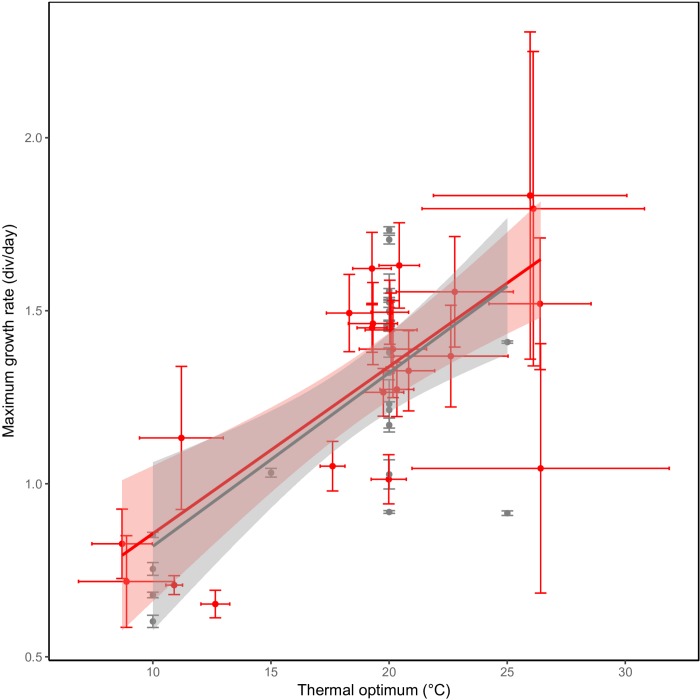
Hotter is better. A higher maximum growth rate in *C. closterium* strains (*y*-axis) is observed in strains with a higher thermal optimum (*x*-axis). The gray points represent the experimentally observed maximum growth rate ( ± SE) and the temperature at which this was observed whilst the red points represent the predicted maximum growth rate with the predict thermal optimum. For both predicted parameters, the standard error (see section “Materials and Methods”) is shown. Linear regressions were fitted to each of the datasets in the respective colors.

Besides differences in optimum temperature and thermal niche width, maximum growth rate also differed between the investigated *C. closterium* strains. The observed maximum growth rate ranges from to 0.6 to 2 divisions per day. The predicted maximum growth rates, based on the growth equations, had a similar range: from 0.65 to 1.8 divisions per day. The observed and predicted rates correlated strongly (Pearson correlation = 0.97, *p* < 0.0001), confirming the reliability of the model. A strong and significant (*p* ≤ 0.001) positive linear relation was observed between the (predicated) maximum growth rates and the thermal optima of strains (Figure 5). On average, an increase of 1°C in thermal optima was accompanied with a maximal growth rate increase of approximately 0.05 divisions d^−1^.

### Cell Viability Under Extreme Temperatures

Application of the SYTOX-Green stain indicated that survival at 0.5°C was generally better compared to exposure at 33°C in the studied *C. closterium* strains (Figure 3). Six out of 8 tested strains (SP01, PT01, CCMP1554, PS10, MID22, and GB01) exhibited 5–18% viable cells after 4 weeks exposure at 0.5°C, indicating that, although growth could not be measured in these isolates, at least part of the population can survive. In contrast, the tropical strain CS-114 and the subtropical strain CS-5, both originating from Australian waters, did not survive at this low temperature (Figure 3).

The temperature effect at 33°C was more severe, since only 2 out of 8 selected strains (H3, MID22) showed 2–8% viable cells after 4 weeks incubation. All other tested *C. closterium* strains did not survive this temperature.

## Discussion

The phylogenetic analysis based on the five loci resulted in a well-resolved phylogeny which exposed the high genetic variability present within *C. closterium* (Figure 2). Based on phylogenetic evidence and mating experiments, Li et al. (2007) and Vanormelingen et al. (2013) already suggested, the existence of a cryptic species complex rather than a single *C. closterium* species. The species delineation results, which indicate the presence of up to 15 different cryptic species in this study alone, confirm these suspicions. Detailed taxonomic studies using morphological analyses and reproductive assays in addition to the molecular information will be required to fully resolve this species complex as was done for other species complexes (Amato et al., 2007; De Decker et al., 2018). Furthermore, an extended sampling effort will probably increase the number of extant species and allow to statistically detect biogeographical patterns such as isolation by distance, if they are present (Casteleyn et al., 2010).

The present study indicates different thermal growth optima for the 24 investigated *C. closterium* strains, with closely related strains displaying similar thermal niches. The thermal niche is largely conserved within species and even among closely related species. The strain-specific temperature requirements for growth indicate that the *C. closterium* species complex consists of numerous genotypes which might explain the ecological success of *C. closterium* in geographically widely distributed habitats. Furthermore, the high genetic diversity among the temperate strains and a polar clade consisting of northern and southern hemisphere strains provides evidence for the high dispersal capability of *C. closterium*. The ability of *C. closterium* to disperse easily to the water column (Araújo et al., 2013), probably promotes dispersal in this species complex.

The obtained thermal optima were in accordance with previous studies. Scholz and Liebezeit (2012) investigated a *C. closterium* strain from a German intertidal mud flat of the North Sea and reported a thermal niche width between 10°C and 25°C which corresponds well with the growth response patterns of most of the temperate strains of the present study. Morris and Kromkamp (2003), however, showed temperature optima for photosynthesis at 30°C in a *C. closterium* isolate from the Ems-Dollard Estuary, Netherlands. This is much higher compared to the growth optima of the 24 *C. closterium* strains investigated in this study, ranging between 9 and 27°C. Temperature optima for photosynthesis, however, can be higher than those for growth because both physiological processes are not directly coupled in algae (Davison, 1991; Eggert and Wiencke, 2000). This indicates that temperature effects on a specific physiological process like photosynthesis do not necessarily match the temperature-growth pattern because growth as a more general physiological process integrates all positive and negative influences of temperature on the whole metabolism and hence better reflects physiological activity and viability (Bulthuis, 1987). The temperature requirements for growth of the Arctic *C. closterium* strain KD10 confirm the results on other benthic diatoms in this biogeographic region (Karsten et al., 2006). Surprisingly, the 4 Antarctic strains of *C. closterium* exhibited a similar thermal response with a relatively broad growth temperature tolerance compared to typical endemic Antarctic benthic diatoms (Longhi et al., 2003). Hence, it is reasonable to assume that they do not carry the same physiological traits as these typical endemics.

Thermal niches were conserved within clades of *C. closterium*, with the majority of the clades growing best around 20°C. It is plausible that there is a stabilizing selection for *C. closterium*, a selection in favor of this intermediate temperature and potentially against extremer temperatures. Under such a scenario, some clades will adapt to thermal extremes, but most will be drawn back toward this more common thermal optimum around 20°C. This is in accordance with the lower, yet significant, Blomberg’s K observed in this study (Ackerly, 2009) and the nestedness of cold-water clade III within temperate clades II and IV.

Very little is known about the role of thermal adaptation in speciation (Keller and Seehausen, 2012). If temperature does play a major role in ecological speciation, we would expect closely related species to have different thermal niches, and a tight association between cladogenesis and divergence in thermal habitats (Svensson, 2012). Such patterns were not observed. Notably, some of the strains belonging to different putative species co-occurred and had a similar thermal niche. This does not rule out the effects of temperature on speciation as we also observed thermal exclusion between several strains: these strains cannot co-exist due to their thermal demands. If not responsible for the speciation itself, thermal adaptation might be relevant in the spatial isolation of species.

Compared to the temperate and (sub)tropical strains, the polar strains had a much narrower thermal performance range. The width of the thermal performance range was only weakly linked to the geographical distribution of the strains and seemed to be more dependent on the physiology of the strains. Strains with a higher thermal optimum and growth rate also tended to have a larger thermal performance range. This is in contrary to the *Jack-of-all-trades is a master-of-non* hypothesis (Huey and Hertz, 1984), which states that the ability to perform at a broad temperature range can only be achieved at the sacrifice of a high growth rate. In other words, the hypothesis claims there is an expected trade-off between performance breadth and maximal performance, which was not the case here. The positive correlation between the maximum growth rate and thermal optimum is, however, in line with the *hotter is better* hypothesis (Huey and Kingsolver, 1989). This hypothesis states that high temperatures inevitably accelerate biochemical reactions and thus growth. Similar patterns have been observed for other organisms (Knies et al., 2009) and will have important implications for the response of *C. closterium* to climate change. In contrast to Thomas et al. (2012) who predicted tropical microalgae are more sensitive to increasing temperatures due to their optima being closer to the currently experienced temperatures, our results suggest that polar strains are the most vulnerable with respect to global change. The much narrower performance range and slower growth rates of the polar strains will be a large handicap when competing with temperate strains under higher temperatures. However, the thermal tolerance of polar strains might evolve as temperatures gradually increase, allowing polar strains to persist (Schaum et al., 2018). It is worth mentioning that predictions on the effects of global change on coastal microalgae remain largely speculative. Climate change will not result in uniform warming of coastal waters (Liao et al., 2015) nor will its effects be limited to temperature alone. Changes in precipitation and evaporation will act on the local salinity and nutrient concentrations (Scavia et al., 2002), which will in turn affect the capability of the algae to respond to temperature fluctuations (Singh and Singh, 2015).

Adaptation to a different temperature regime can be achieved through a combination of different mechanisms. For instance, by increasing the intracellular concentrations of ribulose-1,5-bisphosphate carboxylase/oxygenase (RuBisCO), a key enzyme for photosynthesis, to compensate for its poor catalytic efficiency at low temperatures (Devos et al., 1998). Other adaptive mechanisms include the evolution of cold or heat shock and antifreeze proteins, the modulation of the key enzyme kinetics, and an alternate composition of the biological membranes through the incorporation of different polyunsaturated fatty acid chains (Morgan-Kiss et al., 2006). It is likely that many of these mechanisms are present in *C. closterium*, which would to explain the broad thermal tolerances that was observed for some *C. closterium* strains, allowing them to survive the high temperature fluctuations typical of most coastal habitats. Whole genome sequencing combined with transcriptomics could provide more insight on the presence and relevance of such thermal adaptations (Stillman and Armstrong, 2015; Mock et al., 2017).

Membrane integrity, as an important defense barrier against all environmental influences, is considered as the prerequisite for the cell’s survival, and hence it was reported to be the least stress-sensitive trait of microorganisms (Freese et al., 2006). Here, we showed that strong cell biological damage occurred in many *C. closterium* strains during 4-weeks exposure at very low, but particularly at very high temperatures (Figure 3). The SYTOX-Green assays indicate that the thermal niche width for growth can be confirmed by the membrane properties and hence viability, i.e., lower and upper temperatures for growth, are in close proximity to the thresholds for mortality.

## Conclusion

In conclusion, we have found a large genetic diversity within *C. closterium* which most likely represents multiple (pseudo)cryptic species. The strains in this species complex display diverging thermal responses which might explain the global ecological success of *C. closterium*. Due to the narrower thermal niche and lower maximal growth of polar strains, we foresee a potential replacement of cold-adapted species by those originating from warmer areas as water temperatures continue to rise. Additional research on the structure of the cosmopolitan *C. closterium* complex will provide us with the opportunity to better understand their biogeography and, when combined with information on their thermal response, will help to better predict the effects of climate change on these key organisms of shallow water coastal zones.

## Author Contributions

BV, KS, UK, and WV designed the study. BV performed the growth experiments and isolated several of the strains used in this study. FR performed the SYTOX-Green assays. WS analyzed the data. UK and WS wrote the manuscript which was revised by KS and WV. KS, UK, and WV provided the funding.

## Conflict of Interest Statement

The authors declare that the research was conducted in the absence of any commercial or financial relationships that could be construed as a potential conflict of interest.

## Footnotes

1 http://www.iobis.org

## Supplementary Material

MATERIAL S1 Single locus phylogenies. The phylogenies were constructed with RAxML under the same settings as the concatenated phylogeny. The outgroup IIDO2 was not included for the two most variable markers (*cox*1 and ITS). The clades, as shown by roman numerals in Figure 2, are indicated by colors for ease of comparison between phylogenies. Bootstrap values left of the nodes indicate their support.

MATERIAL S2 Growth of the *C. closterium* strains at the different temperatures. The growth ± SE for each strain is shown at the tested temperatures. The average growth per temperature is displayed in gray. The fitted function is plotted in black and the estimated optimal temperature is shown in red. The thermal performance range, defined as the temperature range at which ≥80% of the predicted maximum growth can be achieved, is delineated by pink lines.
