# Supplementary figures and images for: Thermal Niche Differentiation in the Benthic Diatom Cylindrotheca closterium (Bacillariophyceae) Complex
Source: Front Microbiol. 2019 Jun 21;10:1395. doi: 10.3389/fmicb.2019.01395 (PMC6598499; doi:10.3389/fmicb.2019.01395)

# Supplementary material 1: single locus phylogenies

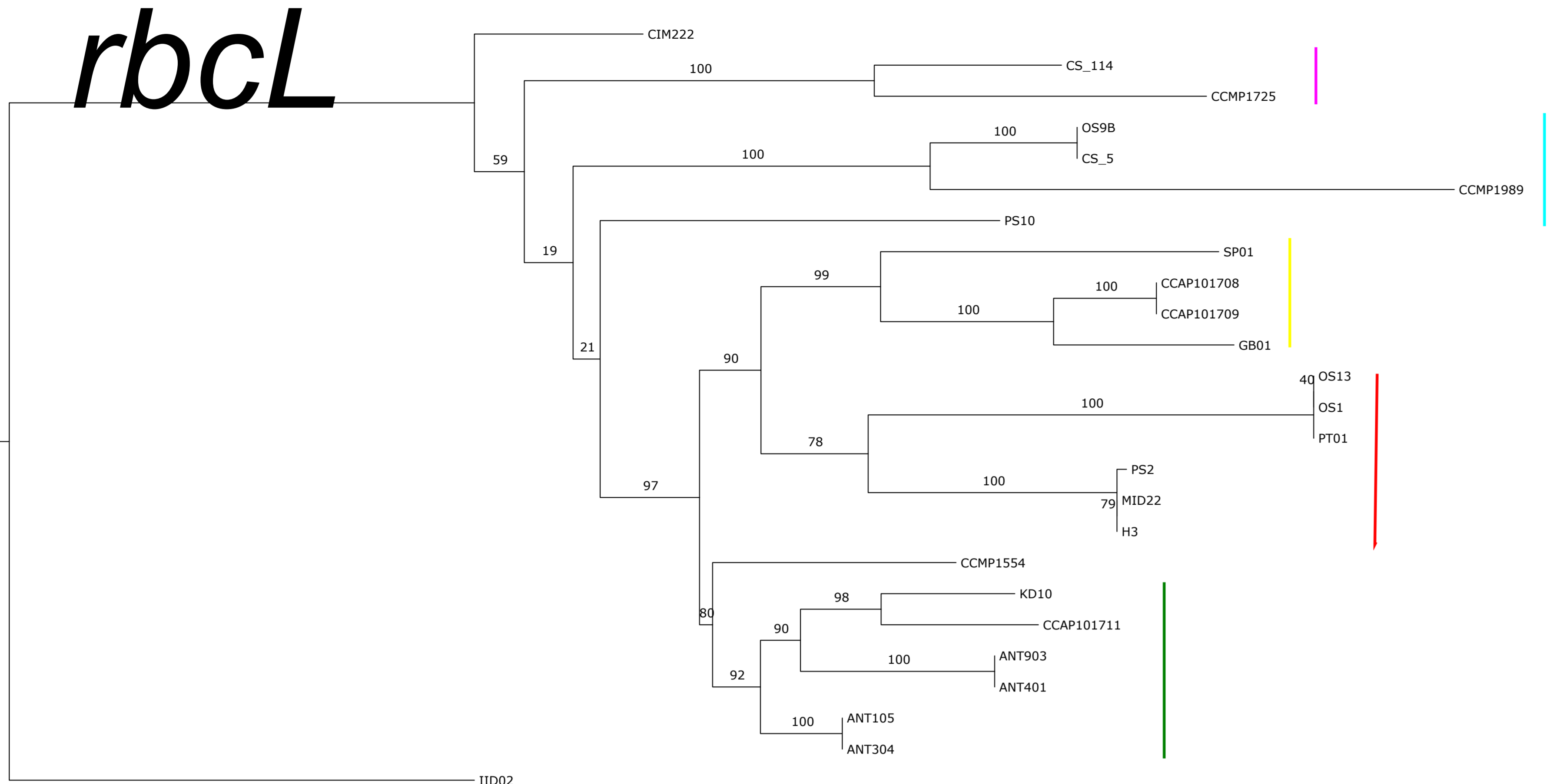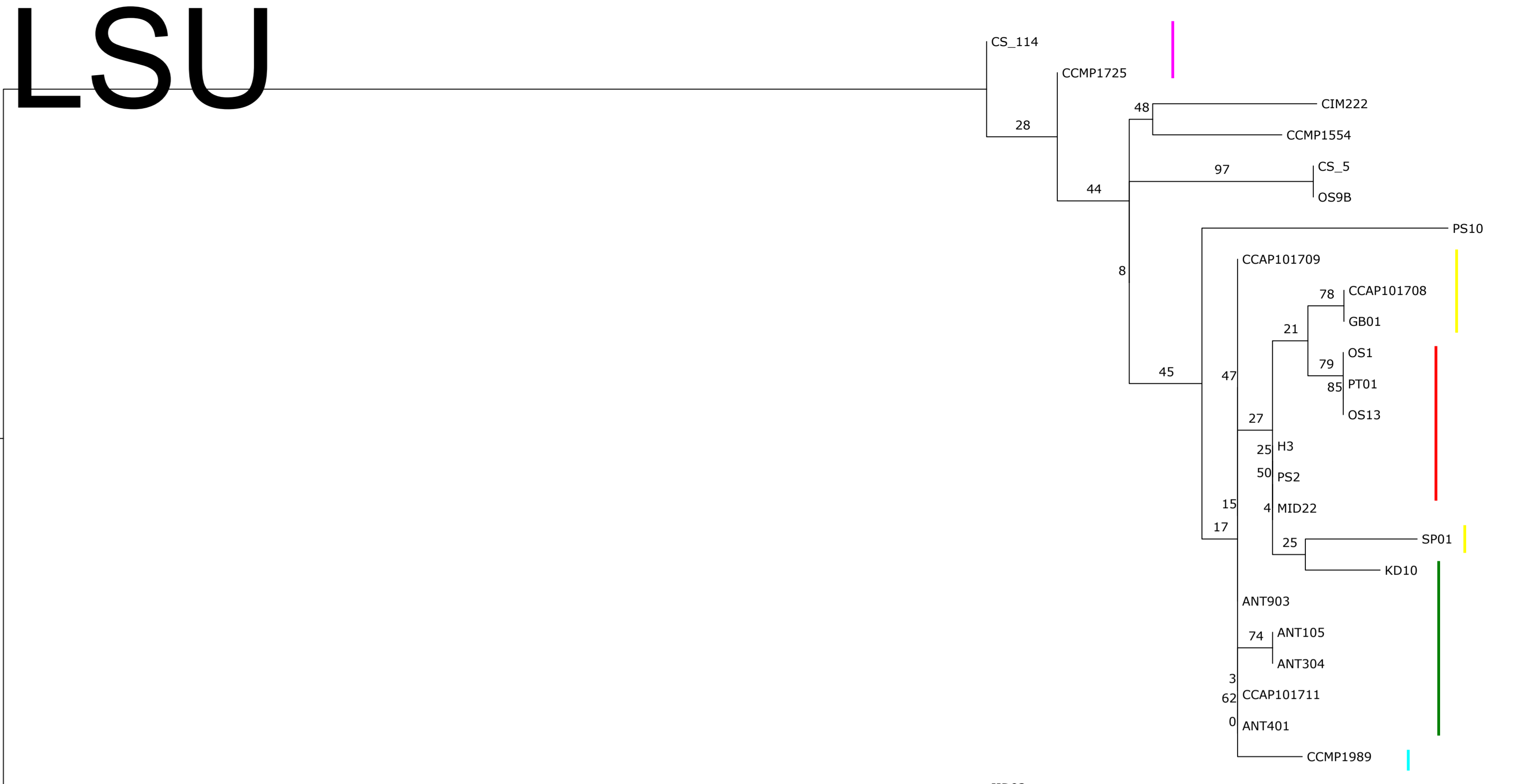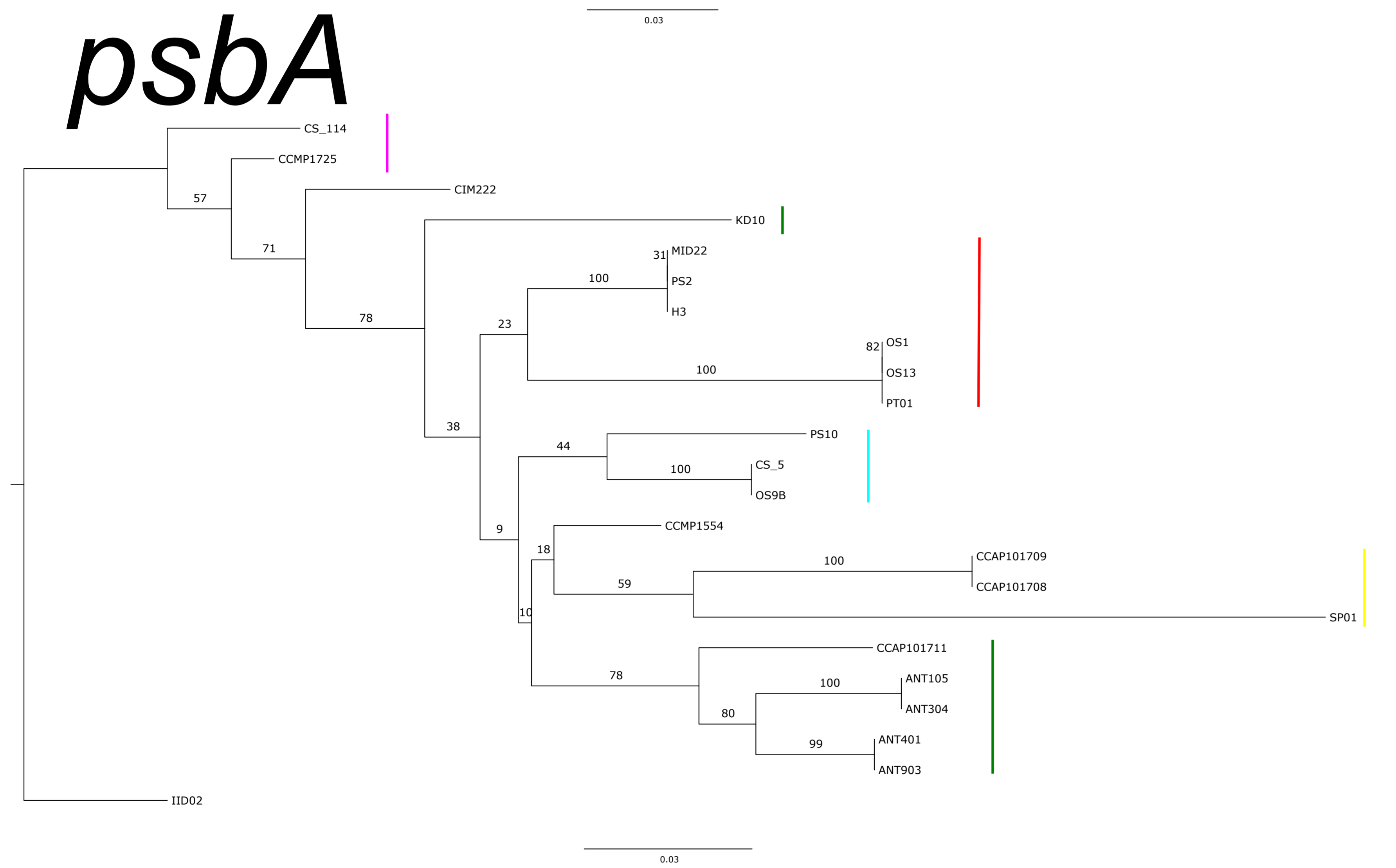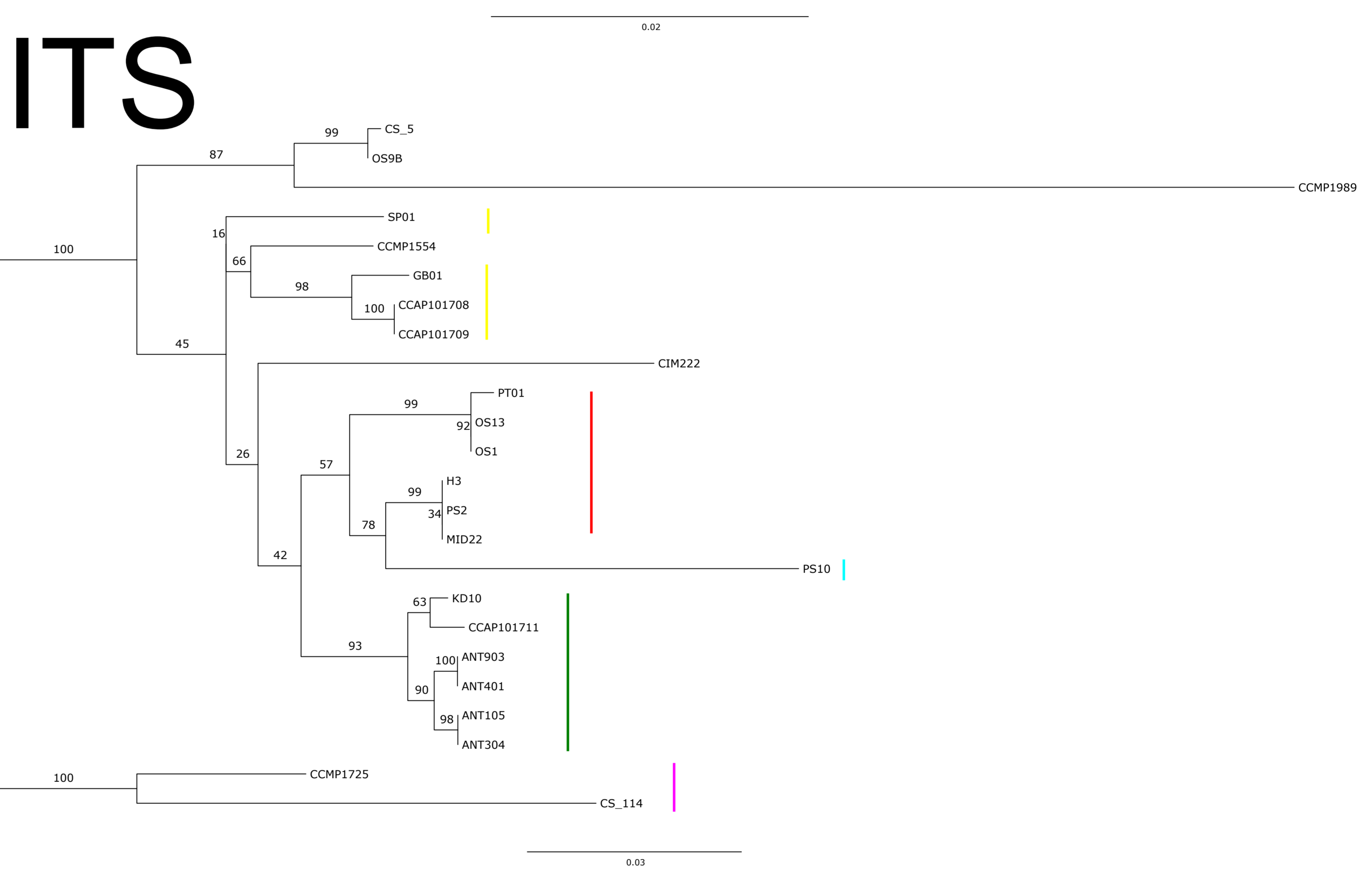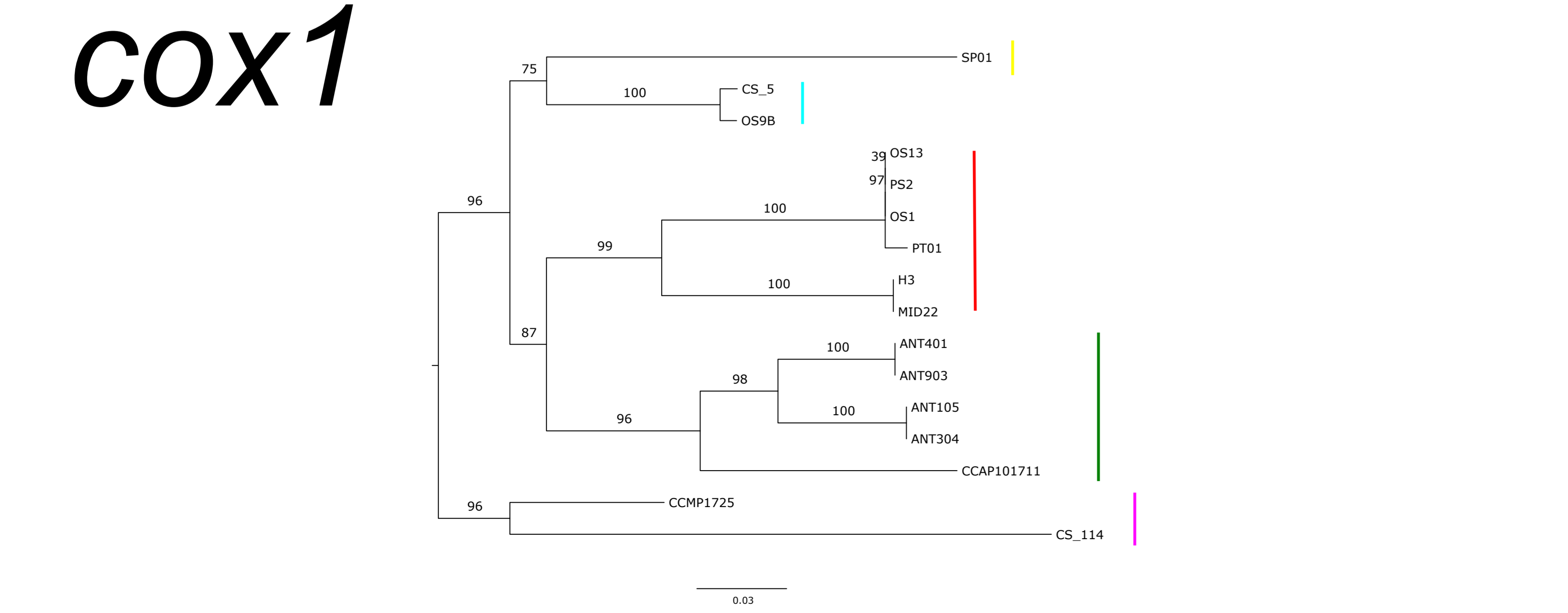

- Clade I
- Clade II
- Clade III
- Clade IV
- Clade V

Supplement: MATERIAL S1 — Single locus phylogenies. The phylogenies were constructed with RAxML under the same settings as the concatenated phylogeny. The outgroup IIDO2 was not included for the two most variable markers (cox1 and ITS). The clades, as shown by roman numerals in Figure 2, are indicated by colors for ease of comparison between phylogenies. Bootstrap values left of the nodes indicate their support. [file Data_Sheet_1.PDF]

**ANT105**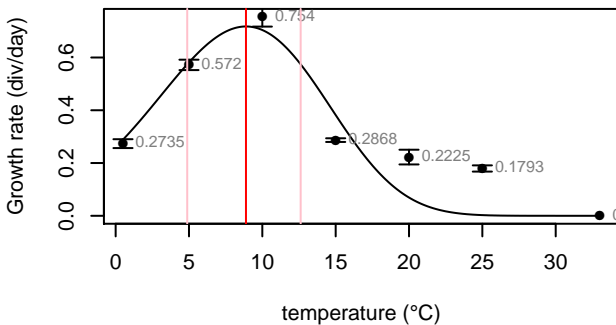**ANT304**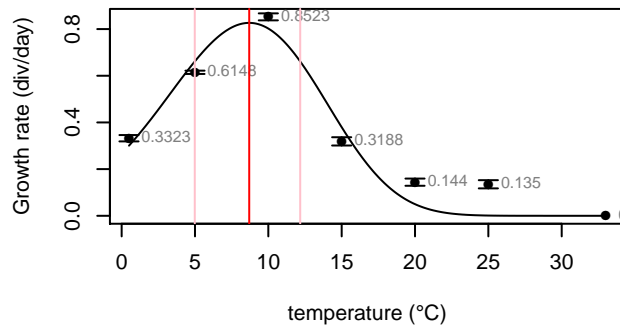**ANT401**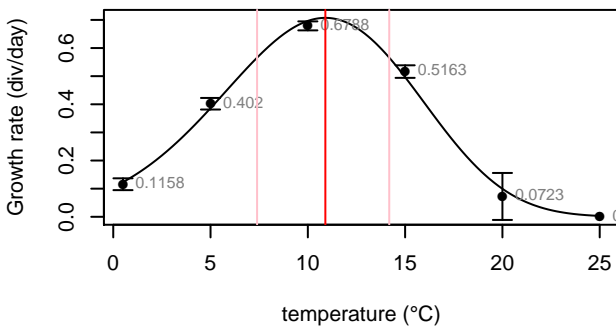**ANT903**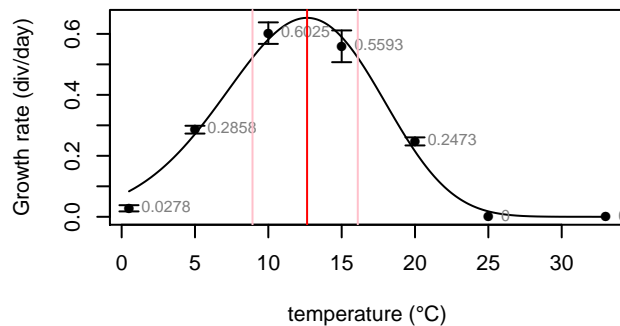**CCAP101708**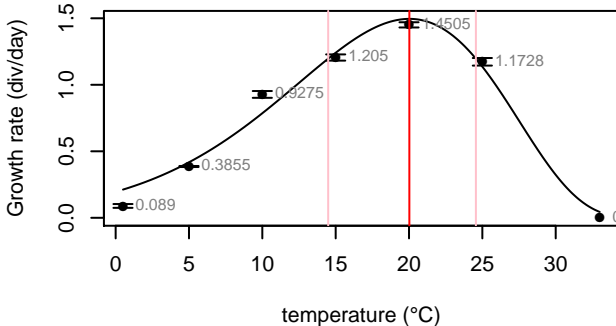**CCAP101709**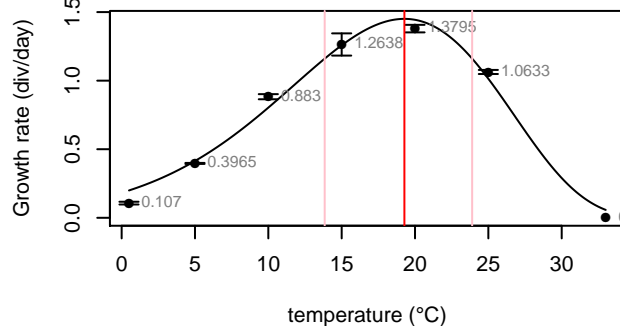

**CCAP101711**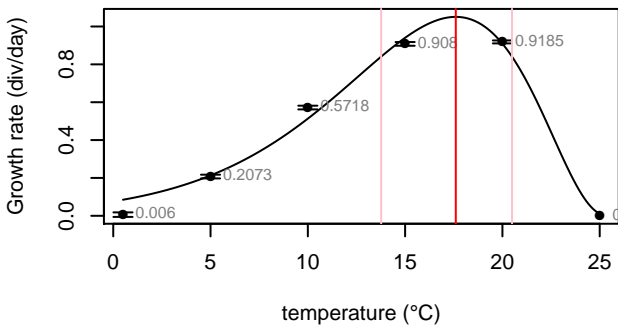**CCMP1554**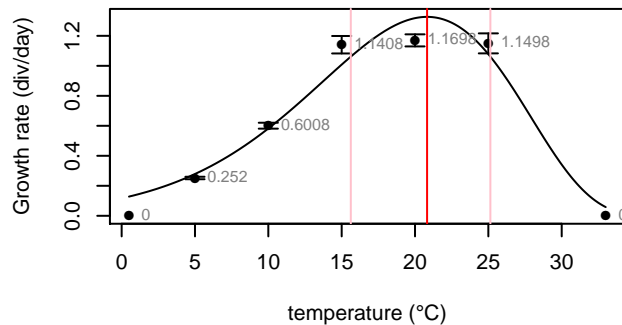**CCMP1725**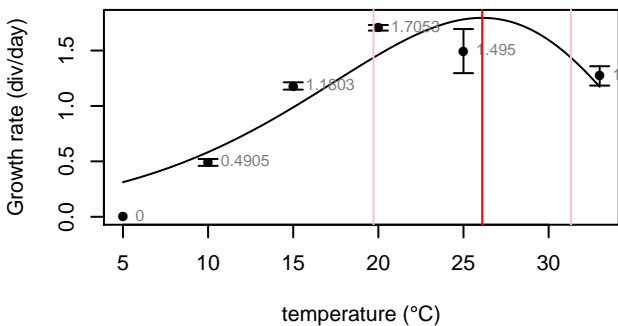**CCMP1989**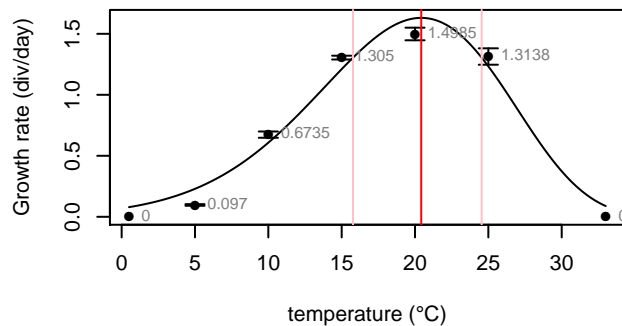**CIM222**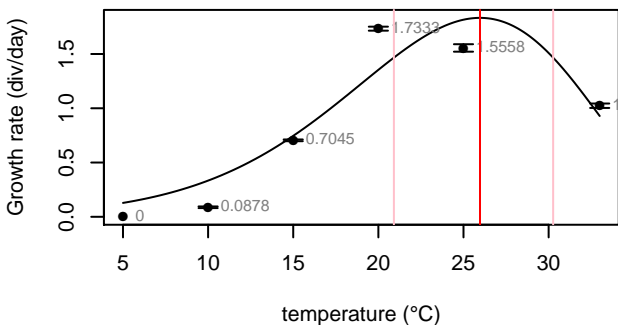**CS\_114**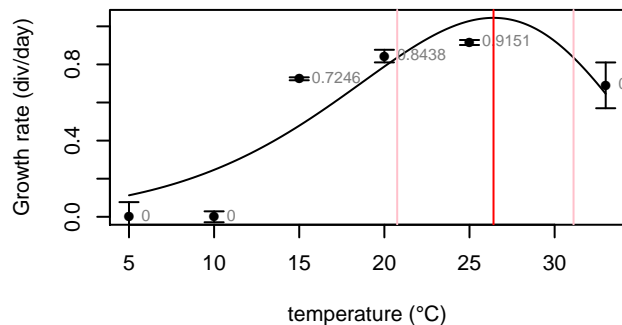

**CS\_5**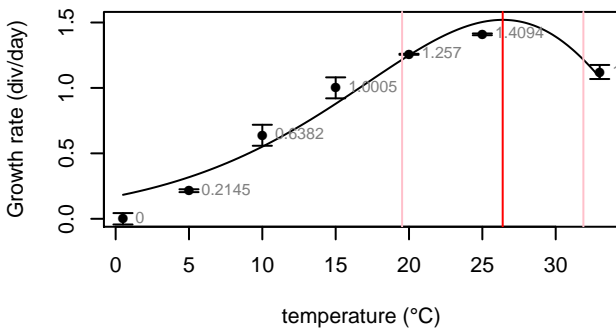**GB01**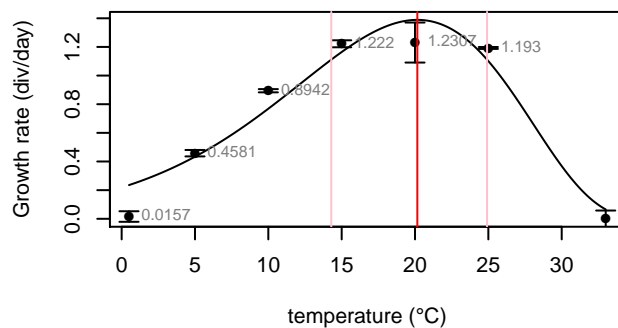**KD10**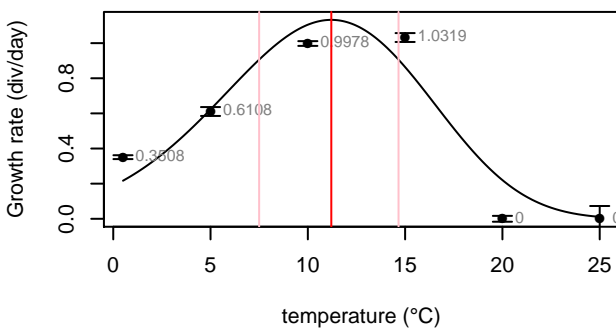**OS1**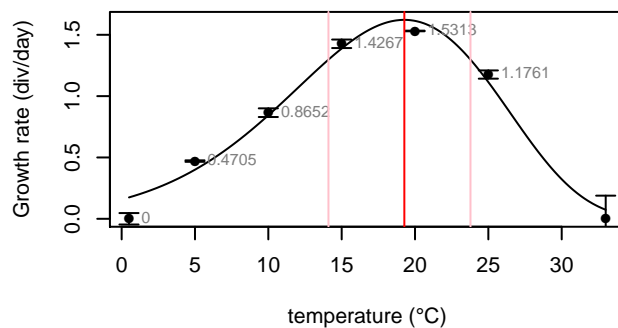**OS13**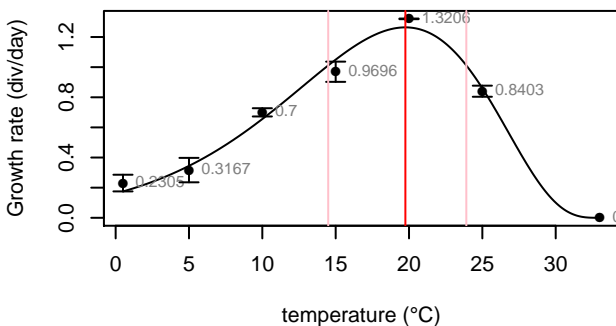**OS9B**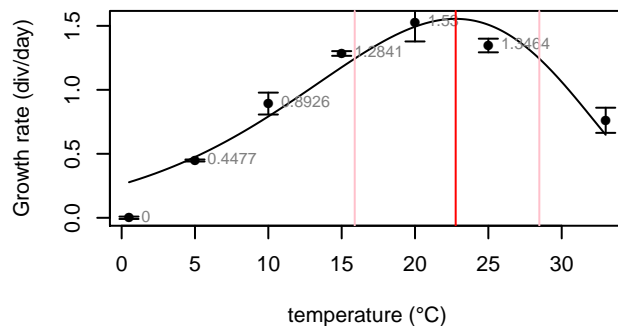

**PS10**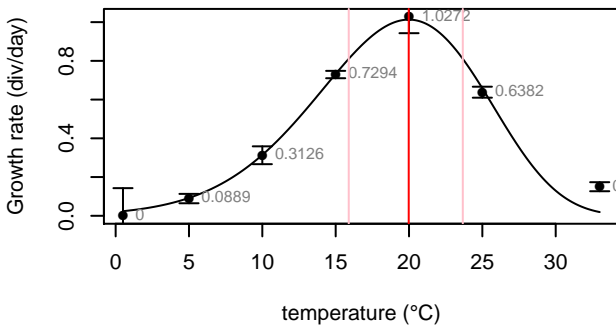**PS2**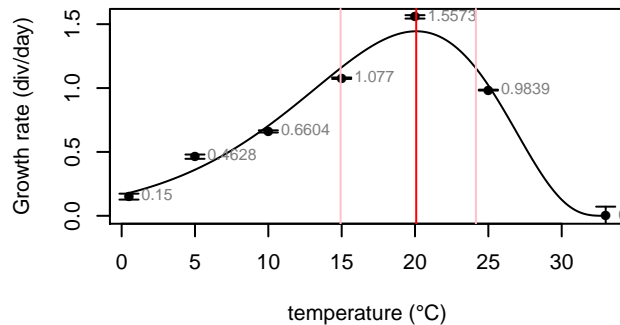**PT01**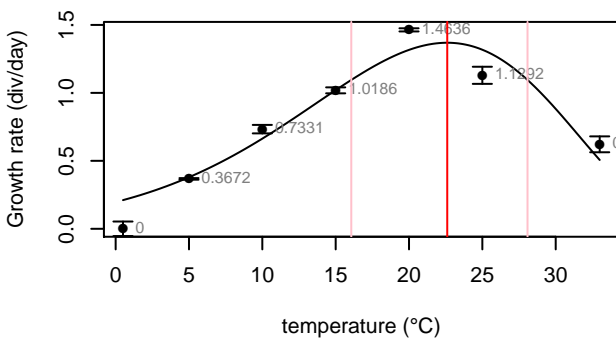**SP01**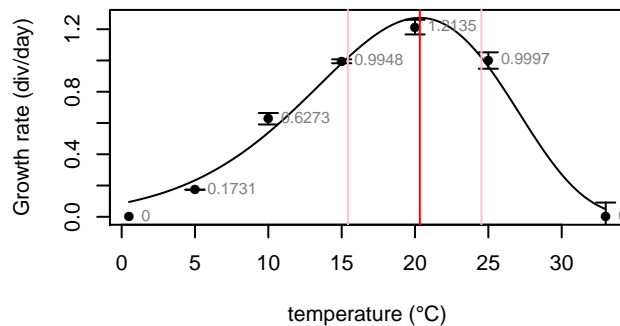

Supplement: MATERIAL S2 — Growth of the C. closterium strains at the different temperatures. The growth ± SE for each strain is shown at the tested temperatures. The average growth per temperature is displayed in gray. The fitted function is plotted in black and the estimated optimal temperature is shown in red. The thermal performance range, defined as the temperature range at which ≥80% of the predicted maximum growth can be achieved, is delineated by pink lines. [file Data_Sheet_2.PDF]
